# The workforce for health in a globalized context – global shortages and international migration

**DOI:** 10.3402/gha.v7.23611

**Published:** 2014-02-13

**Authors:** Christoph Aluttis, Tewabech Bishaw, Martina W. Frank

**Affiliations:** 1 Department of International Health, CAPHRI School for Public Health and Primary Care, Faculty of Health, Medicine and Life Sciences, Maastricht University, Maastricht, The Netherlands; 2 Ethiopian Public Health Association (EPHA), Addis Ababa, Ethiopia; 3 Alliance for Brain Gain and Innovative Development (ABIDE), Addis Ababa, Ethiopia; 4 African Federation of Public Health Associations (AFPHAs), Addis Ababa, Ethiopia; 5 Independent Consultant, Global Health, Zurich, Switzerland

**Keywords:** health workforce migration, global health, globalization, WHO Code of Practice

## Abstract

The ‘crisis in human resources’ in the health sector has been described as one of the most pressing global health issues of our time. The World Health Organization (WHO) estimates that the world faces a global shortage of almost 4.3 million doctors, midwives, nurses, and other healthcare professionals. A global undersupply of these threatens the quality and sustainability of health systems worldwide. This undersupply is concurrent with globalization and the resulting liberalization of markets, which allow health workers to offer their services in countries other than those of their origin. The opportunities of health workers to seek employment abroad has led to a complex migration pattern, characterized by a flow of health professionals from low- to high-income countries. This global migration pattern has sparked a broad international debate about the consequences for health systems worldwide, including questions about sustainability, justice, and global social accountabilities. This article provides a review of this phenomenon and gives an overview of the current scope of health workforce migration patterns. It further focuses on the scientific discourse regarding health workforce migration and its effects on both high- and low-income countries in an interdependent world. The article also reviews the internal and external factors that fuel health worker migration and illustrates how health workforce migration is a classic global health issue of our time. Accordingly, it elaborates on the international community's approach to solving the workforce crisis, focusing in particular on the WHO Code of Practice, established in 2010.

Healthcare services are a rapidly growing sector of the world economy. Globalization processes and the worldwide increase in demand for healthcare have not only fueled the trade in healthcare technologies but also opened domestic borders for foreign labor in the health sector, resulting in cross-border migration of health workers.^1^ Whilnational health workforce migration was discussed as early as 1974 (1), it has dramatically increased in scale due to the liberalization of markets and changes in population dynamics over the past two decades (2). While it is difficult to provide a precise picture of global migratory flows, various studies indicate a pattern that is characterized by migration from low- and middle-income countries (LMICs) to high-income countries (HICs) in North America and Western Europe (3–6).

The freedom of health workers to offer their services in a globalized employment market must be seen within the context of a global undersupply of human resources for health. The World Health Organization (WHO) stated in 2006 that globally there was a shortage of almost 4.3 million doctors, midwives, nurses, and other health workers. It further estimated that globally 75 countries had fewer than 2.5 health workers per 1000 population, which is the ‘minimum number necessary to deliver basic health services’ (3). According to the same report, the large majority of countries with a serious shortage of health workers are located on the African continent. Already stressed health systems in countries such as Zimbabwe, Nigeria, Ghana, Zambia, and South Africa experience a net outflow of health workers, while HICs in North America, Europe, the Middle East, and Oceania actively ‘import’ health service labor in order to sustain their healthcare systems in the light of existing or anticipated shortages (4).

The HICs’ reliance on health workers from overseas contributes to the so-called brain drain phenomenon in LMICs, in which highly skilled personnel leave a particular country in order to offer their services elsewhere. This shift in resources is believed to play out particularly intensely in Africa. According to WHO, Africa has only 10% of the world's population, yet they bear 25% of the global disease burden. This disease burden in turn is confronted by only 3% of the whole global health workforce (3). While the African continent already is a particular hotspot when it comes to shortages in health service delivery, health workforce migration is likely to exacerbate the situation. Depending on the particular profession and country, African countries are estimated to lose up to 70% of their health workforces to HICs. Approximately 65,000 African-born physicians and 70,000 African-born professional nurses are currently working overseas in HICs (4). This lack of health workers on the African continent can literally create life-endangering situations for communities where the health services simply vanish overnight due to the emigration of qualified personnel. Especially in sub-Saharan countries, health worker migration is believed to have serious negative effects on the availability and quality of health services (5–7). Furthermore, it has been claimed that LMICs not only lose manpower in the health sector, but also effectively lose out on their financial investments into training and education (5, 6).

## Health workforce flow in a globalized context

Adequately quantifying the flows and stocks of health workers in a globalized world is a very difficult endeavor, as reliable information is nearly impossible to obtain, and is generally described as of poor quality (7) or even anecdotal (8). The difficulties in establishing accurate data on global workforce flows stem from, among other factors, a lack of registration data in both sending and receiving countries, the complexities of migration pathways, and the definition of a migrating health worker's status in the receiving country (i.e. whether the migration is temporary or permanent) (7–9). The lack of reliable data therefore makes it very difficult to sketch an accurate picture of workforce migration patterns. One sophisticated attempt to map and quantify health workforce flows was conducted in 2007 by the Organisation for Economic Co-operation and Development (OECD), which reviewed the in- and outflow of doctors and nurses for OECD countries based on the best available data. Regarding OECD members which are also members of the European Union (EU), the authors estimated that the percentage of foreign-born nurses ranged from 0.4% in Finland to 25.8% in Luxembourg. In Switzerland, which is not a member of the EU-28, this number rises to 28.6%. For doctors, the percentages ranged from 3.2% in Poland to 35.3% in Ireland (8). A full overview of selected countries from Europe that are included in the OECD study can be found in Table 1. In addition, the study showed that over the past 25 years, the number and the percentage of foreign-trained nurses and doctors increased significantly in European countries (8). Making a distinction between *foreign-born* and *foreign-trained* health professionals is important, as it is the foreign-*trained* health workers that are of relevance in the discussion on migration and the related shortages of health workers—and not the foreign-*born* professionals, who may have gotten their degree in the country of their current residence.

**Table 1:** Number and percentage of foreign-trained nurses and doctors in European OECD countries

	Nurses			Doctors		
		
Country of residence	Total	Foreign born	% Total	Total	Foreign born	% Total
Austria	56,797	8, 217	14.5	30,068	4,400	14.6
Belgium	127,384	8,409	6.6	39,133	4,629	11.8
Denmark	57,047	2,320	4.1	14,977	1,629	10.9
Finland	56,365	470	0.8	14,560	575	4.0
France	421,602	23,308	5.5	200,358	33,879	16.9
Germany	781,300	74,990	10.4	282,124	28,494	11.1
Greece	39,952	3,883	9.7	13,744	1,181	8.6
Hungary	49,738	1,538	3.1	24,671	2,724	11.0
Ireland	43,320	6,204	14.3	8,208	2,895	35.3
Luxembourg	2,551	658	25.8	882	266	30.2
Netherlands	259,569	17,780	6.9	42,313	7,032	16.7
Norway	70,698	4,281	6.1	12,761	2,117	16.6
Poland	243,225	1,074	0.4	99,687	3,144	3.2
Portugal	36,595	5,077	13.9	23,131	4,552	19.7
Spain	167,498	5,638	3.4	126,248	9,433	7.5
Sweden	98,505	8,710	8.9	26,983	6,148	22.9
Switzerland	62,194	17,636	28.6	23,039	6,431	28.1
United Kingdom	538,647	81,623	15.2	147,677	49,780	33.7

Source: OECD (8).

The OECD study showed that nearly all European OECD countries increasingly rely on recruiting health workers from abroad to fill their shortages. The Health Professional Mobility in the European Union Study (PROMeTHEUS) points in the same direction as the OECD findings and estimates that countries such as Estonia, Slovakia, and Poland have little reliance on foreign medical doctors, with a demand ranging from 0.02 to 0.7% of the total workforce. On the contrary, countries like Switzerland, Slovenia, Ireland, and the United Kingdom were found to be among the European countries with very high reliance on foreign medical doctors, with 22.5 to 36.8% of their current workforce having been trained abroad (10). Both OECD and PROMeTHEUS studies express that migration occurs heavily between European countries. For example, most of Ireland's foreign-trained nurses were trained in the United Kingdom. In Norway, the large majority of migrant nurses come from other Scandinavian countries. Unfortunately, the immigration of doctors and nurses from non-European countries can hardly be assessed systematically, as data on the exact flows of personnel are very limited and difficult to obtain. This prevents us from establishing a clear picture of the flow of health workers between European countries and the rest of the world. Nevertheless, in a study of 10 EU member states, Dussault, Fronteira, and Cabral estimate that, on average, one-third of migrant doctors came from outside the European Union. This figure rises to 60% in France and Italy, and even to 80% in both Ireland and the United Kingdom (11).

### Push and pull factors

A wealth of contemporary literature is concerned with reviewing the so-called push and pull factors that determine whether a person will remain in their country of training or work elsewhere (12–14). *Push factors* are internal in nature, and they are those factors that exist in the country of origin and drive a health worker away from the health system in which they were trained. *Pull factors* in turn are external, because they describe those circumstances in the destination countries which provide an incentive for health workers to immigrate. Recent systematic literature reviews highlighted that various financial, professional, political, social, and personal factors can act as both push and pull factors that contribute to health workers’ decisions to migrate. These factors include better remuneration in other countries, professional advancement and better career opportunities, a safer and better working environment, and a better quality of life (12). Also political factors, such as getting away from unstable regions, can act as push factors and play a major role in decisions to leave the country (14). Clemens found that there is a positive correlation between greater political stability and prosperity, on one hand, and health worker retention, on the other (4). Alternatively, civil war and economic stagnation were strong predictors for health worker emigration (11). Additional push factors can include extremely unsatisfactory working conditions in the country of origin, lack of medicine and inadequate supplies and equipment, a large nurse-to-patient ratio, and epidemics of HIV/AIDS and other serious illnesses, which contribute to making work stressful in developing countries (13, 14).

The high global demand for health workers and the liberalized global market are further revealed by a variety of employment organizations in HICs which pursue active recruitment strategies in LMICs. It has been estimated that in the United States alone, 270 companies were engaged in international nurse recruitment, a sharp increase from approximately 40 companies that existed in the late 1990s (15). This, despite the fact that overseas recruitment has been widely described as unjust, unethical, and even criminal by some (16, 17).

## Cost and benefits of workforce migration

There is a broad consensus in the scientific literature that health worker migration has negative effects on the sending country and its people, while the receiving country and the health worker will benefit. However, the picture is somewhat more complex, and any review should include all possible factors that can be considered beneficial or detrimental. A comprehensive overview of the costs and benefits of health worker migration can be found in Table 2, which complements a previous review of Stewart, Clark, and Clark (18) and provides an overview of the relevant claims in the current discussion. The authors suggest that in principle there are two groups who benefit most from health workforce migration: the migrants themselves and the residents of the recipient country (18). At first, health workers themselves clearly benefit due to usually better working conditions, better career opportunities, and higher salaries. In addition, the residents of the recipient country benefit from an adequate supply of healthcare services and a savings of tax moneys through training fewer healthcare professionals than they would otherwise need (19). Some argue that the sending countries benefit to some extent as well from receiving financial remittances from the health workers living in developed countries (20, 21). However, this view has not been uncontested, as remittances throughout the whole life course can hardly match the training and education costs invested in the migrant (5, 22). Furthermore, doctors and nurses receive publicly financed education, whereas remittances are usually sent back to families in private. Remittances therefore may even have a negative effect of exacerbating inequalities by increasing the wealth of the privileged while impoverishing the poor (22). Others argue that in the case of circular migration patterns, countries can gain significant skills and knowledge if the emigrant worker returns home after a few years abroad. One survey found that 50% of physicians in the United Kingdom who emigrated from an LIC had the intention to return to their home countries at some point (23). The underlying assumption here is that these individuals will have enhanced knowledge and skills that they can put to use once they return to their home country. Another article further points to the complex pathways involved in calculating financial costs and benefits of migration and states that migration could -under certain circumstances- have a positive effect on the welfare of the country of origin (24). Contrary to this, a study by Mills et al. of nine sub-Saharan countries asserts that the collective investment loss for training those doctors who are currently working abroad approaches $2.17 billion (7). Reportedly, Kenya alone loses an investment of about US$500,000 for every doctor who migrates. For each emigrating nurse, Kenya loses investments worth US$300,000 (6). Accordingly, the gains of these investments shift to the receiving countries, who can save on their training investments. It has been estimated that the total financial savings of recruiting doctors from abroad amounted to up to $2.7 billion for the United Kingdom and $846 million for the United States, thereby effectively acting as a subsidy for HIC health systems (7).

**Table 2:** Costs and benefits in sending and receiving countries

Effects of health worker migration	Sending countries	Receiving countries
Costs	• Shortages in domestic healthcare service capacity	• Some administrative costs involved
	• Financial loss in investment of training and educating the workforce	• Enhanced local competition
	• Financial loss of consumption and tax receipts	
	• Decline in morale and commitment among remaining workers	
	• Loss of social and human capital	
	• Knowledge spillover losses	
	• Undermining institution building and development as a whole	
	• Loss of expert knowledge in academia and education centers	
	• Loss of role models for young students	
Benefits	• Remittances received from people working abroad	• Relief of supply shortages
	• Improvements in skills of returnees	• Improved quality of healthcare
	• Collaborative partnership between diaspora and local professionals	• Tax receipts from foreign workers

Source: Adopted from Stewart, Clark, and Clark (18) and further enhanced.

In addition to the economic dimensions, the sustainability of healthcare systems in developing countries increasingly comes under pressure as facilities become understaffed, the quality of care decreases, and the morale among the remaining staff deteriorates. With regard to the effect of migration on direct health outcomes, only a few studies exist that try to link health outcomes with migration, as it is very difficult to establish a causal relationship. One notable study showed that in countries in which the HIV prevalence rate exceeds 3%, a doubling of the medical brain drain rate was associated with a 20% increase in adult deaths from HIV/AIDS (25). However, the currently existing scientific evidence is insufficient to make any sound statements on the direct health effects of health worker migration. However, it seems common sense that losing health workers will lead to a deterioration of quality of health services, which will ultimately lead to more negative health outcomes (21).

### WHO's Code of Practice

This review has shown that migration of health workers is a phenomenon in which decisions and actions taken in one country can have substantial and direct effects on another country. Recruiting doctors and nurses from a LMIC to serve the demand in HICs effectively creates a shortage in the country of origin, and hence contributes to worse health outcomes. Finding solutions to this problem therefore can occur only at the international level, as the problem's determinants often cross borders and governments. From a global health governance perspective, one of the most notable actions to tackle the health workforce crisis was formulated in 2010 during WHO's 63rd World Health Assembly. The ‘Global Code of Practice on the International Recruitment of Health Personnel’ intends to serve as a policy framework for addressing the health workforce crisis at a global scale. It establishes a framework for the ethical recruitment of health personnel and guides its member states in the development of national frameworks for ethical recruiting (26). The Code of Practice proposes that conditions for the recruitment of health personnel should be set out in bilateral agreements between source and destination countries, thereby creating win-win situations in the context of health workforce migration. These bilateral agreements could, for instance, foresee a reimbursement of the source country for every migrating doctor. But yet again, this would require systematic and effective monitoring of migratory flows. The Code further stresses that it respects the rights of individual health workers to migrate and therefore asks source countries to address the factors that drive the health worker's emigration. This is an important point, as the individual right of a migrant to seek opportunities elsewhere can conflict with the country's goals to secure the provision of health services of its people. Improving health worker retention while at the same time respecting their individual rights can be achieved by improving working conditions in the donor country itself. In return, the destination countries are particularly asked by the Code to ensure adequate and context-specific long-term health workforce planning, focusing on capacity building of local professionals in order to decrease the pressure to ‘import’ health workers from elsewhere. Furthermore, destination countries are asked to support sending countries technically and financially to mitigate the current effects of migration of health personnel (26). Such solutions to the workforce crisis have been widely discussed and considered effective in tackling the shortages of health workers (27).

The Code of Practice can be viewed as a laudable initiative, as it identifies a global understanding of the problem at hand, and functions as an early attempt to coordinate international cooperation across health systems. Fidler even describes the Code as part of a series of ‘groundbreaking governance regimes’ for the global health problems of our time (28). However, as with many international agreements, one of the Code's major weaknesses is that it is voluntary in nature to its signatories. Member states and other stakeholders are therefore merely encouraged to apply the Code to their national practices, and compliance to the Code is hardly monitored or enforced. This raises the question of whether the current Code is sufficiently powerful to change countries’ behavior toward more ethical recruitment guidelines. In this regard, some notable suggestions have been made to improve its effectiveness and to alleviate the health workforce crisis. First of all, as the lack of data substantially hampers the effective implementation of any workforce policies, Glinos et al. call for a systematic monitoring of migratory flows in order to understand whether policies and strategies suggested by the Code are being respected and effective. Even HICs across Europe struggle in monitoring the in- and outflow of migrant health workers (29). Addressing this issue is key to successfully developing policies and interventions to meet the problems caused by health workforce migration. Secondly, Glinos et al. suggest the introduction of national accountability frameworks that ensure compliance with the Code, including compliance from public administration and healthcare providers. Such accountability frameworks could ensure compliance at the national level, as they could include sanctions or fines for non-compliance (29). Despite these certainly useful recommendations, it remains questionable whether HICs will move toward this direction. Currently, it is simply too easy (and financially lucrative) for HICs to secure their undersupply of health professionals by importing foreign staff. Incentives to change the current policies under the Code of Practice are therefore relatively small, and the current global power relations seem to be skewed in such a way that many HICs benefit from the current practice, which makes them likely to resist any actions that put restrictions on them.

## Summary and conclusion

The phenomenon of health workforce migration can be labeled a classic global health issue of our time. Globalization fuels migration, and health workers worldwide are becoming increasingly mobile, connected, and aware of the opportunities in other, more affluent countries. While there is still a lack of systematic monitoring of migratory flows, existing studies show that a global market for health professionals has developed, leading to a global increase of doctors and nurses migrating to other countries. However, from a health needs perspective, this global market for doctors, nurses, and other health professionals appears to be distorted. In a globalized market, HICs can address their shortages in health personnel by recruiting and importing qualified health workers from elsewhere. Shortages thereby are shifted from HICs to LMICs, thereby increasing global inequities. Because many of the so-called pull factors are external in nature (i.e. outside a country's regulatory boundaries), a LMIC can do little to influence these factors in order to prevent the emigration of its qualified health workforce.

This article has discussed several issues around this problem. It showed how the shift of labor can have both positive and negative implications, depending on the country's role in the migratory scheme. While the migrating health workers and the receiving countries benefit in general, it is the donor countries and its citizens who suffer most from the brain drain. While health worker migration is not desirable in terms of healthcare service quality and equity, it also reveals a structural problem of healthcare systems worldwide. The chronic global undersupply of health workers points to the fact that many HIC healthcare systems are unsustainable, as they rely on foreign labor in order to provide their services. In many EU countries, sufficient provision of health workers already depends on immigration. In the light of aging societies and projected future increases in the demand for health workers, this current status seems unsustainable. Even worse, in times of economic crisis, countries less affected by recession could attract even more health professionals from countries where salaries in the medical sector are being cut and the health workforce is being downsized. The current practice by many HICs to fill their staff needs by recruiting therefore merely serves as a ‘quick fix’ and cloaks unsustainable practices, as the underlying problems of undersupply are not tackled effectively. For European countries, it should be apparent that recruiting health workers from overseas does not solve the global workforce crisis; it only shifts shortages to a country that is even less well situated to cope with the shortages, potentially inducing life-threatening situations in those countries. But as long as the international demand outweighs its supply, training more health workers in low-income countries will not be an effective solution, as this simply serves to further fuel the export market. The WHO Code of Practice has been a small step in the right direction. However, the only realistic and sustainable policy option can be to simply create a greater supply of health workers in HICs by means of increasing the training capacities, improving overall working conditions, and making the jobs of nursing and other healthcare professionals more attractive to the domestic workforce. While this is not the easiest and cheapest solution, it is the only one that can effectively tackle the global undersupply while also being fair and sustainable to LMICs.

## Footnotes

1 A health worker is defined as ‘anyone engaged in actions whose primary intent is to enhance health’ (3). In this article, however, we limit our analysis to doctors and nurses, as data are mainly available for these two professions (3). When we refer to health workers, we therefore address doctors and nurses – unless otherwise specified.
